# Supplementary material for: Clinical improvement in canine pulmonary hypertension with Perna canaliculus oil (PCSO-524) add-on therapy: Effects on exercise tolerance and cough
Source: PLoS One. 2025 Sep 29;20(9):e0333526. doi: 10.1371/journal.pone.0333526 (PMC12478914; doi:10.1371/journal.pone.0333526)
Supplement: S1 Table — (DOCX) [file pone.0333526.s001.docx]

**S1 Table:** Ordinal scoring system for quality of life and respiratory variables.

| **Variable** | **Score** | **Clinical Correlate** |
| --- | --- | --- |
| Exercise tolerance | 1 (Very good) | Dog moved around with ease, was able to fully exercise |
|  | 2 (Good) | Dog moved around with ease, was not able to fully exercise; ability to run was reduced |
|  | 3 (Moderate) | Dog was less active than normal, moved around a few times per day, avoided long walks |
|  | 4 (Poor) | Dog was inactive and would only get up to eat, drink, or urinate |
| Demeanor | 1 | Alert, responsive |
|  | 2 | Mildly depressed |
|  | 3 | Moderately depressed |
|  | 4 | Minimally responsive |
|  | 5 | Unresponsive |
| Appetite | 1 | Increased |
|  | 2 | Normal |
|  | 3 | Decreased (2/3 normal) |
|  | 4 | Markedly decreased (<2/3 normal) |
| Respiratory effort | 1 | Normal |
|  | 2 | Mildly increased rate or effort |
|  | 3 | Moderately labored |
|  | 4 | Severe respiratory distress |
| Coughing | 1 | None |
|  | 2 | Occasional (a few times a week) |
|  | 3 | Frequent (a few times a day) |
|  | 4 | Persistent (frequently during the day) |
| Nocturnal dyspnea/cough | 1 | None |
|  | 2 | Dog coughed from time to time during the night, but no other clinical signs of dyspnea or restlessness were present |
|  | 3 | Dog coughed consistently; increased respiratory effort or restlessness during the night |

Source: Haggstrom et al. (2008)^25^

25. Häggström J, Boswood A, O'Grady M, Jons O, Smith S, Swift S, et al. Effect of Pimobendan or Benazepril Hydrochloride on Survival Times in Dogs with Congestive Heart Failure Caused by Naturally Occurring Myxomatous Mitral Valve Disease: The QUEST Study. J Vet Intern Med. 2008. PubMed PMID: 18638016.
