# Supplementary material for: Clinical improvement in canine pulmonary hypertension with Perna canaliculus oil (PCSO-524) add-on therapy: Effects on exercise tolerance and cough
Source: PLoS One. 2025 Sep 29;20(9):e0333526. doi: 10.1371/journal.pone.0333526 (PMC12478914; doi:10.1371/journal.pone.0333526)
Supplement: S2 Table — (DOCX) [file pone.0333526.s002.docx]

**S2 Table**: Medications prescribed before administration of sildenafil, PCSO**-**524, and placebo. Doses are illustrated as mg/kg/day.

| **Medications** |  | **Frequencies and doses of the PCSO-524 group** | | |  | **Frequencies and doses of the placebo group** | | | **P** |
| --- | --- | --- | --- | --- | --- | --- | --- | --- | --- |
|  | n | Mean ± SD | Min | Max | n | Mean ± SD | Min | Max |  |
| Furosemide | 6 | 4.04 ± 1.59 | 1.33 | 5.64 | 8 | 3.7 ± 1.80 | 1.63 | 7.7 | 0.72 |
| Enalapril | 2 | 0.75 ± 0.07 | 0.70 | 0.80 | 4 | 0.81 ± 0.22 | 0.60 | 1.0 | 0.74 |
| Ramipril | 6 | 0.18 ± 0.04 | 0.14 | 0.25 | 5 | 0.20 ± 0.04 | 0.15 | 0.24 | 0.33 |
| Pimobendan | 5 | 0.46 ± 0.06 | 0.40 | 0.54 | 7 | 0.42 ± 0.09 | 0.30 | 0.50 | 0.34 |
| Aminophylline | 2 | 16.97 ± 6.74 | 12.20 | 21.74 | 0 | - | - | - | NA |
| Spironolactone | 1 | NA | 1.76 | - | 0 | - | - | - | NA |

Comparisons between groups were tested by independent T-test.
