# Supplementary material for: Clinical improvement in canine pulmonary hypertension with Perna canaliculus oil (PCSO-524) add-on therapy: Effects on exercise tolerance and cough
Source: PLoS One. 2025 Sep 29;20(9):e0333526. doi: 10.1371/journal.pone.0333526 (PMC12478914; doi:10.1371/journal.pone.0333526)
Supplement: S4 Table — (DOCX) [file pone.0333526.s004.docx]

**S4 Table:** Distribution of continuous variables of all 3 visits (visit 1 = day 0, visit 2 = day 35, and visit 3 = day 70) of all 17 dogs. Results are shown as mean ± standard deviation (SD) for normally distributed variables and median with interquartile ranges for non-normally distributed.

| **Variables/visits** | **Mean ± SD or median (inerquartile)** | **P model** | **P (different between visits**  **within subjects)** | | |
| --- | --- | --- | --- | --- | --- |
|  |  |  | **1 vs 2** | **1 vs 3** | **2 vs 3** |
| Age (years) | 12.1 **±** 3.1 | NA |  |  |  |
| Body weight (kg) |  | 0.45 | 1.00 | 1.00 | 0.69 |
| Visit 1 | 5.0 (3.8, 6.2) |  |  |  |  |
| Visit 2 | 5.0 (3.9, 6.1) |  |  |  |  |
| Visit 3 | 5.0 (3.7, 6.0) |  |  |  |  |
| Body temperature (F) |  | 0.84 | 0.90 | 0.84 | 0.99 |
| Visit 1 | 100.9 **±** 0.8 |  |  |  |  |
| Visit 2 | 101.0 **±** 0.8 |  |  |  |  |
| Visit 3 | 101.1 **±** 0.8 |  |  |  |  |
| HR-PE (bpm) |  | 0.89 | 1.00 | 1.00 | 1.00 |
| Visit 1 | 130 (130, 140) |  |  |  |  |
| Visit 2 | 136 (126, 140) |  |  |  |  |
| Visit 3 | 120 (120, 142) |  |  |  |  |
| Respiratory rate (BrPM) |  | 0.21 | 1.00 | 0.31 | **1.00** |
| Visit 1 | 28 (24, 35) |  |  |  |  |
| Visit 2 | 28 (24, 32) |  |  |  |  |
| Visit 3 | 28 (24, 31) |  |  |  |  |
| SBP (mmHg) |  | 0.13 | 0.26 | 0.31 | 1.00 |
| Visit 1 | 152 (140, 182) |  |  |  |  |
| Visit 2 | 160 (140, 183) |  |  |  |  |
| Visit 3 | 150 (144, 163) |  |  |  |  |
|  |  |  |  |  |  |
|  |  |  |  |  |  |
| VVTI |  | 0.74 | 0.76 | 0.79 | 1.00 |
| Visit 1 | 7.90 ± 2.02 |  |  |  |  |
| Visit 2 | 8.27 ± 1.81 |  |  |  |  |
| Visit 3 | 8.24 **±** 2.33 |  |  |  |  |
| HR-ECG (bpm) |  | 0.07 | 0.08 | 0.95 | 0.16 |
| Visit 1 | 140 ± 27 |  |  |  |  |
| Visit 2 | 127 ± 26 |  |  |  |  |
| Visit 3 | 138 **±** 21 |  |  |  |  |
| LA/Ao |  | 0.75 | 1.00 | 1.00 | 1.00 |
| Visit 1 | 1.96 (1.72, 2.45) |  |  |  |  |
| Visit 2 | 1.96 (1.62, 2.63) |  |  |  |  |
| Visit 3 | 2.00 (1.70, 2.24) |  |  |  |  |
| LVIDDN |  | 0.21 | 0.78 | 0.19 | 0.51 |
| Visit 1 | 1.73 ± 0.39 |  |  |  |  |
| Visit 2 | 1.76 ± 0.34 |  |  |  |  |
| Visit 3 | 1.81 ± 0.38 |  |  |  |  |
| LVIDSN |  | 0.78 | 1.00 | 0.82 | 0.81 |
| Visit 1 | 0.82 ± 0.27 |  |  |  |  |
| Visit 2 | 0.82 ± 0.23 |  |  |  |  |
| Visit 3 | 0.84 ± 0.24 |  |  |  |  |
| %FS |  | 0.92 | **0.94** | 0.93 | **1.00** |
| Visit 1 | 51.5 ± 9.0 |  |  |  |  |
| Visit 2 | 52.1 ± 8.0 |  |  |  |  |
| Visit 3 | 52.1 ± 9.1 |  |  |  |  |
| EPSS |  | 0.65 | 1.00 | 1.00 | 1.00 |
| Visit 1 | 1.34 (0.26, 1.96) |  |  |  |  |
| Visit 2 | 1.10 (0.34, 2.00) |  |  |  |  |
| Visit 3 | 1.37 (0.93, 1.90) |  |  |  |  |
|  |  |  |  |  |  |
| E wave (m/s) |  | 0.40 | 0.43 | 1.00 | 0.50 |
| Visit 1 | 1.4 ± 0.5 |  |  |  |  |
| Visit 2 | 1.3 ± 0.5 |  |  |  |  |
| Visit 3 | 1.4 ± 0.6 |  |  |  |  |
| A wave (m/s) |  | 0.15 | 0.24 | 0.17 | 0.98 |
| Visit 1 | 0.9 ± 0.3 |  |  |  |  |
| Visit 2 | 1.0 ± 0.4 |  |  |  |  |
| Visit 3 | 1.0 ± 0.3 |  |  |  |  |
| E/A ratio |  | 0.24 | 0.69 | 0.31 | **1.00** |
| Visit 1 | 1.61 (1.21, 2.03) |  |  |  |  |
| Visit 2 | 1.45 (1.10, 1.63) |  |  |  |  |
| Visit 3 | 1.37 (1.02, 1.72) |  |  |  |  |
| MR (mmHg) |  | 0.24 | 0.22 | 0.50 | 0.84 |
| Visit 1 | 117.4 ± 33.5 |  |  |  |  |
| Visit 2 | 126.4 ± 31.5 |  |  |  |  |
| Visit 3 | 123.4 ± 26.2 |  |  |  |  |
| TR (mmHg) |  | ***0.001*** | 0.05 | ***0.008*** | 0.25 |
| Visit 1 | 73.4 ± 27.8 |  |  |  |  |
| Visit 2 | 65.8 ± 28.2 |  |  |  |  |
| Visit 3 | 60.7 ± 23.8 |  |  |  |  |
| Total protein (g/dL) |  | 0.36 | 0.74 | 0.75 | 0.33 |
| Visit 1 | 7.0 ± 0.7 |  |  |  |  |
| Visit 2 | 6.8 ± 2.0 |  |  |  |  |
| Visit 3 | 7.3 ± 0.5 |  |  |  |  |
| Albumin (g/dL) |  | 0.73 | 1.00 | 1.00 | 1.00 |
| Visit 1 | 3.0 (2.8, 3.3) |  |  |  |  |
| Visit 2 | 3.1 (2.9, 3.3) |  |  |  |  |
| Visit 3 | 3.1 (3.0, 3.4) |  |  |  |  |
|  |  |  |  |  |  |
| ALP (iu/L) |  | 0.39 | 0.60 | 1.00 | 0.91 |
| Visit 1 | 120 (62, 242) |  |  |  |  |
| Visit 2 | 106 (61, 206) |  |  |  |  |
| Visit 3 | 123 (54, 210) |  |  |  |  |
| ALT (iu/L) |  | 0.31 | 0.79 | 0.43 | 1.00 |
| Visit 1 | 82 (45, 114) |  |  |  |  |
| Visit 2 | 71 (48, 96) |  |  |  |  |
| Visit 3 | 64 (39, 98) |  |  |  |  |
| BUN (mg/dL) |  | 0.90 | 1.00 | 1.00 | 1.00 |
| Visit 1 | 27 (22, 42) |  |  |  |  |
| Visit 2 | 28 (22, 34) |  |  |  |  |
| Visit 3 | 31 (21, 45) |  |  |  |  |
| Creatinine (mg/dL) |  | 0.66 | 1.00 | 1.00 | 1.00 |
| Visit 1 | 1.2 (0.9, 1.6) |  |  |  |  |
| Visit 2 | 1.3 (0.9, 1.7) |  |  |  |  |
| Visit 3 | 1.2 (0.9, 1.7) |  |  |  |  |
| RBC (10^12^/L) |  | 0.85 | 0.93 | 0.98 | 0.84 |
| Visit 1 | 6.7 ± 1.1 |  |  |  |  |
| Visit 2 | 6.6 ± 1.3 |  |  |  |  |
| Visit 3 | 6.7 ± 1.3 |  |  |  |  |
| PCV (%) |  | 0.86 | 0.85 | 0.92 | 0.99 |
| Visit 1 | 45.6 ± 7.2 |  |  |  |  |
| Visit 2 | 45.0 ± 8.5 |  |  |  |  |
| Visit 3 | 45.2 ± 8.2 |  |  |  |  |
| Hemoglobin (g/dL) |  | 0.70 | 0.67 | 0.89 | 0.92 |
| Visit 1 | 15.4 ± 2.4 |  |  |  |  |
| Visit 2 | 15.2 ± 2.7 |  |  |  |  |
| Visit 3 | 15.3 ± 2.8 |  |  |  |  |
|  |  |  |  |  |  |
| MCV (fL) |  | 0.90 | 0.96 | 0.98 | 0.89 |
| Visit 1 | 68.6 ± 3.0 |  |  |  |  |
| Visit 2 | 68.3 ± 3.4 |  |  |  |  |
| Visit 3 | 68.8 ± 6.2 |  |  |  |  |
| MCH (g/dL) |  | 0.40 | 0.76 | 0.36 | 0.79 |
| Visit 1 | 23.2 ± 1.2 |  |  |  |  |
| Visit 2 | 23.1 ± 1.4 |  |  |  |  |
| Visit 3 | 22.9 ± 1.4 |  |  |  |  |
| MCHC (g/dL) |  | 0.83 | 0.81 | 0.95 | 0.95 |
| Visit 1 | 34.0 ± 1.6 |  |  |  |  |
| Visit 2 | 33.8 ± 0.8 |  |  |  |  |
| Visit 3 | 33.9 ± 0.9 |  |  |  |  |
| Platelet (10^9^/L) |  | 0.50 | 0.54 | 1.00 | 0.58 |
| Visit 1 | 413.1 ± 143.1 |  |  |  |  |
| Visit 2 | 393.7 ± 192.3 |  |  |  |  |
| Visit 3 | 411.8 ± 156.1 |  |  |  |  |
| RDW (%) |  | 0.77 | 0.88 | 0.76 | 0.97 |
| Visit 1 | 13.3 ± 1.3 |  |  |  |  |
| Visit 2 | 13.4 ± 1.3 |  |  |  |  |
| Visit 3 | 13.5 ± 1.3 |  |  |  |  |
| Plasma protein (g/dL) |  | 0.91 | 1.00 | 0.90 | 0.94 |
| Visit 1 | 8.9 ± 0.8 |  |  |  |  |
| Visit 2 | 9.0 ± 0.9 |  |  |  |  |
| Visit 3 | 9.0 ± 0.9 |  |  |  |  |
| WBC (106/L) |  | 0.97 | 1.00 | 0.98 | 0.96 |
| Visit 1 | 12,730 ± 5,151 |  |  |  |  |
| Visit 2 | 12,613 ± 5,144 |  |  |  |  |
| Visit 3 | 12,951 ± 6,913 |  |  |  |  |
|  |  |  |  |  |  |
| Neutrophils (106/L) |  | 0.86 | 0.94 | 0.97 | 0.84 |
| Visit 1 | 9,911 ± 4,364 |  |  |  |  |
| Visit 2 | 9,535 ± 4,367 |  |  |  |  |
| Visit 3 | 10,175 ± 5,988 |  |  |  |  |
| Lymphocytes (106/L) |  | 0.25 | 026 | 0.94 | **0.42** |
| Visit 1 | 1,886 ± 717 |  |  |  |  |
| Visit 2 | 2,294 ± 1,233 |  |  |  |  |
| Visit 3 | 1,917 ± 866 |  |  |  |  |
| Eosinophils (106/L) |  | 0.14 | 0.12 | 0.51 | 0.64 |
| Visit 1 | 389 ± 301 |  |  |  |  |
| Visit 2 | 243 ± 234 |  |  |  |  |
| Visit 3 | 309 ± 221 |  |  |  |  |
| Monocytes (106/L) |  | 0.84 | 1.00 | 1.00 | 1.00 |
| Visit 1 | 337 (214, 898) |  |  |  |  |
| Visit 2 | 378 (202, 860) |  |  |  |  |
| Visit 3 | 347 (233, 669) |  |  |  |  |
| Neutrophils/lymphocytes ratio |  | 0.11 | 0.12 | 0.60 | 1.00 |
| Visit 1 | 5.49 ± 1.87 |  |  |  |  |
| Visit 2 | 5.62 ± 6.81 |  |  |  |  |
| Visit 3 | 5.29 ± 2.07 |  |  |  |  |
| NT-proBNP (pmol/L) |  | 0.46 | 1.00 | 0.69 | 1.00 |
| Visit 1 | 1,425 (813, 3,074) |  |  |  |  |
| Visit 2 | 1,131 (710, 3,134) |  |  |  |  |
| Visit 3 | 1,310 (640, 3,663) |  |  |  |  |

Comparisons between visits within subjects each group were tested by repeated measured general linear model with pairwise Dunn’s test for normally distributed variables or Friedman with pairwise Wilcoxon signed-rank test for non-normal distributed variables.

Significantly different between each visit are in bold, and italic.

HR-PE: heart rate measured from physical examination; SBP: systolic blood pressure; bpm: beats per minute; BPM: breaths per minute; VVTI: vasovagal tonus index; HR-ECG: heart rate measured from electrocardiography; LA/Ao: left atrial to aortic ratio; LVIDDN: normalized left ventricular internal diameter in diastole; LVIDSN: normalized left ventricular internal diameter in systole; %FS: % fractional shortening; E to A ratio: peak of E wave to peak of A wave ratio; EPSS: E-point septal separation; MR: mitral regurgitation; TR: tricuspid regurgitation; ALP: alkaline phosphatase; ALT: alanine transferase; BUN: blood urea nitrogen; RBC: red blood cell count; PCV: packed cell volume; MCV: mean corpuscular volume; MCH: mean cell hemoglobin; MCHC: mean corpuscular hemoglobin concentration; RDW: red cell distribution width; WBC: white blood cell count; NT-proBNP: N-terminal probrain natriuretic peptide.
