# Supplementary material for: Clinical improvement in canine pulmonary hypertension with Perna canaliculus oil (PCSO-524) add-on therapy: Effects on exercise tolerance and cough
Source: PLoS One. 2025 Sep 29;20(9):e0333526. doi: 10.1371/journal.pone.0333526 (PMC12478914; doi:10.1371/journal.pone.0333526)
Supplement: S5 Table — (DOCX) [file pone.0333526.s005.docx]

**S5 Table**: Comparisons within subjects of the quality of life and respiratory variables scores of all 17 dogs (medians (interquartile)) of all 3 visits (visit 1 = day 0, visit 2 = day 35, and visit 3 = day 70).

| **Variables/visits** | **Median (25th, 75th percentile)** | **P model** | **P (different between visits**  **within subjects)** | | |
| --- | --- | --- | --- | --- | --- |
|  |  |  | **1 vs 2** | **1 vs 3** | **2 vs 3** |
| Exercise tolerance |  | 0.17 | 0.66 | 0.37 | 0.28 |
| Visit 1 | 2 (2, 2) |  |  |  |  |
| Visit 2 | 2 (2, 2) |  |  |  |  |
| Visit 3 | 2 (1, 2) |  |  |  |  |
| Demeanor |  | 0.42 | 0.16 | 0.26 | 0.70 |
| Visit 1 | 1 (1, 2) |  |  |  |  |
| Visit 2 | 1 (1, 1) |  |  |  |  |
| Visit 3 | 1 (1, 1) |  |  |  |  |
| Appetite |  | 0.63 | 0.41 | 0.52 | 0.79 |
| Visit 1 | 2 (2, 2) |  |  |  |  |
| Visit 2 | 2 (2, 2) |  |  |  |  |
| Visit 3 | 2 (2, 2) |  |  |  |  |
| Respiratory effort |  | 0.52 | 0.70 | 0.25 | 0.32 |
| Visit 1 | 2 (1, 2) |  |  |  |  |
| Visit 2 | 2 (1, 2) |  |  |  |  |
| Visit 3 | 2 (1, 2) |  |  |  |  |
| Coughing |  | 0.85 | 0.28 | 0.41 | 0.75 |
| Visit 1 | 2 (2, 2) |  |  |  |  |
| Visit 2 | 2 (2, 2) |  |  |  |  |
| Visit 3 | 2 (2, 2) |  |  |  |  |
| Nocturnal dyspnea/cough |  | 0.87 | 1.00 | 0.70 | 0.56 |
| Visit 1 | 2 (1, 2) |  |  |  |  |
| Visit 2 | 2 (1, 2) |  |  |  |  |
| Visit 3 | 2 (1, 2) |  |  |  |  |

Comparisons between visits within subjects were tested by Friedman with pairwise Wilcoxon signed-rank test.
