# Supplementary material for: Clinical improvement in canine pulmonary hypertension with Perna canaliculus oil (PCSO-524) add-on therapy: Effects on exercise tolerance and cough
Source: PLoS One. 2025 Sep 29;20(9):e0333526. doi: 10.1371/journal.pone.0333526 (PMC12478914; doi:10.1371/journal.pone.0333526)
Supplement: Attachment — Submitted filename: Review 55.pdf [file pone.0333526.s007.pdf]

Title: Clinical Improvement in Canine Pulmonary Hypertension with Perna canaliculus Oil (PCSO-524) Add-on Therapy: Effects on Exercise Tolerance and Cough

Journal: PLOS One

Reviewer: Seyed Amirhossein Mazhari

Reviewer's report:

This randomized study evaluated the effects of PCSO-524, as an add-on therapy for dogs with PH already taking sildenafil and standard care. Seventeen dogs were monitored over 70 days and, the intervention group given PCSO-524 experienced greater improvements in exercise tolerance and reduced coughing compared to the placebo group, suggesting that PCSO-524 may help ease key symptoms of PH in dogs.

At the end I have some questions about this article.

The specific questions and recommendations are as follows:

1:
The manuscript would benefit from a structured abstract.
2:
It is recommended to crate a section for the Ethical considerations. Under Ethical considerations section, it is required to provide more information in the standard format.
3:
I recommend creating a new section for the study limitations.
4:
It is advised that a new section explaining any acronyms used in the article be added at the end, following the conclusion section.

Thanks to the authors for their hard work on this Study. It’s a pleasure to review such a contribution to the field.
