# Supplementary material for: Clinical improvement in canine pulmonary hypertension with Perna canaliculus oil (PCSO-524) add-on therapy: Effects on exercise tolerance and cough
Source: PLoS One. 2025 Sep 29;20(9):e0333526. doi: 10.1371/journal.pone.0333526 (PMC12478914; doi:10.1371/journal.pone.0333526)
Supplement: Attachment — Submitted filename: Review 55a.pdf [file pone.0333526.s010.pdf]

Title: Clinical Improvement in Canine Pulmonary Hypertension with Perna canaliculus Oil (PCSO-524) Add-on Therapy: Effects on Exercise Tolerance and Cough

Journal: PLOS One

Reviewer: Seyed Amirhossein Mazhari

Reviewer's report:

This randomized study evaluated the effects of PCSO-524, as an add-on therapy for dogs with PH already taking sildenafil and standard care. Seventeen dogs were monitored over 70 days and, the intervention group given PCSO-524 experienced greater improvements in exercise tolerance and reduced coughing compared to the placebo group, suggesting that PCSO-524 may help ease key symptoms of PH in dogs.

At the end I have some questions about this article.

The specific questions and recommendations are as follows:

1:
The manuscript would benefit from a structured abstract.

The revised version has the same unstructured abstract as the primary submission.

2:
It is recommended to crate a section for the Ethical considerations. Under Ethical considerations section, it is required to provide more information in the standard format.

It is better presented in the revised version.

3:
I recommend creating a new section for the study limitations.

Has not been addressed by authors.

4:
It is advised that a new section explaining any acronyms used in the article be added at the end, following the conclusion section.

No action by authors.

Thanks to the authors for their hard work on this Revision. It's a pleasure to review such a contribution to the field.
