# Supplementary material for: Clinical improvement in canine pulmonary hypertension with Perna canaliculus oil (PCSO-524) add-on therapy: Effects on exercise tolerance and cough
Source: PLoS One. 2025 Sep 29;20(9):e0333526. doi: 10.1371/journal.pone.0333526 (PMC12478914; doi:10.1371/journal.pone.0333526)
Supplement: Attachment — Submitted filename: Review 55b.pdf [file pone.0333526.s012.pdf]

Title: Clinical Improvement in Canine Pulmonary Hypertension with Perna canaliculus Oil (PCSO-524) Add-on Therapy: Effects on Exercise Tolerance and Cough

Journal: PLOS One

Reviewer: Seyed Amirhossein Mazhari

Reviewer's report:

This randomized study evaluated the effects of PCSO-524, as an add-on therapy for dogs with PH already taking sildenafil and standard care. Seventeen dogs were monitored over 70 days and, the intervention group given PCSO-524 experienced greater improvements in exercise tolerance and reduced coughing compared to the placebo group, suggesting that PCSO-524 may help ease key symptoms of PH in dogs.

All comments and suggestions have been implemented into the second revision.

Thanks to the authors for their hard work on this Revision. It's a pleasure to review such a contribution to the field.
