# Supplementary material for: Clinical improvement in canine pulmonary hypertension with Perna canaliculus oil (PCSO-524) add-on therapy: Effects on exercise tolerance and cough
Source: PLoS One. 2025 Sep 29;20(9):e0333526. doi: 10.1371/journal.pone.0333526 (PMC12478914; doi:10.1371/journal.pone.0333526)
Supplement: Attachment — Submitted filename: review report.docx [file pone.0333526.s008.docx]

Intro

Line 54: PH specific ( spelling mistake)

Line 290: the pancutation is not correct

Line 162: use the both groups instead of 2 groups.

Methods:

-The randomization process could be described more precisely, including how subjects were allocated.

-The data collection points (days 0, 28, and 56) are clearly stated, but justification for the choice of time intervals could be added.

-It would be beneficial to provide more details on how questionnaire reliability was ensured, particularly in assessing clinical signs reported by owners.

Discussion:

-The lack of dietary reporting is appropriately noted but could be expanded with a recommendation for future studies to standardize omega-3 intake.

-The neutrophil-to-lymphocyte ratio and RDW findings are interesting but require a stronger clinical interpretation—whether these changes might have unintended consequences long-term
