# Supplementary material for: Clinical improvement in canine pulmonary hypertension with Perna canaliculus oil (PCSO-524) add-on therapy: Effects on exercise tolerance and cough
Source: PLoS One. 2025 Sep 29;20(9):e0333526. doi: 10.1371/journal.pone.0333526 (PMC12478914; doi:10.1371/journal.pone.0333526)
Supplement: Attachment — Submitted filename: Response to Reviewers.docx [file pone.0333526.s011.docx]

**Significant changes**

To facilitate the review process for the editor and reviewers, we have attached two versions of the manuscript: a Revised Manuscript with Track Changes and a Clean Manuscript.

**Title:**

We confirm the tile “Clinical improvement in canine pulmonary hypertension with *Perna canaliculus* oil (PCSO-524) add-on therapy: effects on exercise tolerance and cough.”

The title has been revised to adhere to *PLOS ONE*'s guidelines for sentence case, as requested. We have ensured that only the first word of the title, proper nouns, and genus names are capitalized.

**Abstract:**

We have completely rewritten the abstract to improve its brevity, flow, and focus. The revised version clearly outlines the objective, methods, key results, and their significance in a coherent narrative under the 300-word limit.

**Introduction**

We have revised the Introduction to better articulate the research gap and the clinical rationale for seeking an adjunctive therapy for PH. The transitions between ideas have been smoothed, and the mechanistic discussion of PCSO-524 has been made more concise and clinically focused.

Materials and Methods

We have performed a significant revision of the manuscript, specifically:

- We have included precise details on the randomization sequence generation (specifying the use of a computer-based random number generator for each severity block) and the allocation concealment process (detailing the use of sequentially numbered, opaque, sealed envelopes).
- we have specified that while a full orthopedic exam was not part of the screening protocol, dogs receiving specific ongoing medication for orthopedic disease (e.g., NSAIDs) were excluded to avoid confounding analgesic effects.

**Results**

We have significantly improved the clarity and professionalism of the Results section, specifically:

- Clarity and Structure: The section has been rewritten with clear subheadings (Study population and baseline characteristics, Between-group comparisons at follow-up, and Within-group changes over time) to improve readability and narrative flow.
- Consistent Terminology: As suggested, all time-point references have been standardized to "Day 0," "Day 28," and "Day 56" throughout the text for improved consistency and precision.
- Correction of Errors: The section has been carefully proofread, and all typographical and grammatical errors, including the specific example of "rricuspid" being corrected to "tricuspid," have been fixed.
- Precise Scientific Language: Vague phrasing has been revised for accuracy. As specifically recommended, the phrase "numerically different improvement" has been replaced with the standard scientific term "a non-significant trend" to describe the finding for exercise tolerance (P=0.06).

**Discussion and Conclusion**

We have performed a thorough rewrite of both the Discussion and Conclusion, specifically:

- Narrative Focus and Clarity: We have restructured the Discussion to present a more logical and coherent narrative. The text now begins with the primary positive findings, followed by a focused interpretation of each key result (clinical scores, TRPG, biomarkers) with improved transitions between ideas.
- Elimination of Redundancy and Imprecision: We have performed a major language edit to eliminate redundancies, awkward phrasing, and imprecise statements. The reasoning for each claim has been clarified.
- Use of References: We have reviewed the use of references to human studies to ensure that their relevance to the canine context is more clearly and directly drawn.
- Significantly expanded the Limitations section; to provide a transparent and detailed discussion of all suggested issues.
- Rewritten Conclusion: The Conclusion has been completely rewritten to be more confident, concise, and impactful. It now starts by stating the primary finding, emphasizes the statistically significant clinical improvements, and ends with a clear and strong recommendation for future research, as suggested.

**Declaration**

***Funding***

As the funder had no role in the study, we have added the required disclaimer to the Declarations section. Furthermore, we have updated the Funding section to read: “This study was funded by the Pharmalink International Ltd., Central, Hong Kong. The funder had no role in study design, data collection and analysis, decision to publish, or preparation of the manuscript.”

**Point-by-point response to each reviewer comment**

*Response for Reviewer #1*

Reviewer reports:

This is an interesting and well-designed study that evaluates the potential benefits of *Perna canaliculus* oil (PCSO-524) as an add-on therapy in dogs with pulmonary hypertension. The topic is relevant to clinical veterinary medicine, especially for practitioners managing canine cardiac patients.

Strengths of the manuscript:

- The prospective, randomized, placebo-controlled trial design adds scientific strength and reliability to the findings.
- The methods, particularly the use of standardized clinical scoring and echocardiographic assessment, are appropriately selected.
- The writing is generally clear and well-organized.
- Ethical approval and owner consent were obtained and clearly reported.

Response: We sincerely thank the reviewer for your time and for your positive and encouraging feedback. We are pleased that you found our study to be well-designed, interesting, and clinically relevant. We appreciate your acknowledgment of the study's strengths, including its methodology and clear reporting.

Suggestions for improvement:

1. Sample size: The study had a relatively small number of dogs (n=17), which limits the generalizability of the findings. Please acknowledge this limitation more clearly in the discussion.
2. Dietary influence: Since omega-3 intake from the dogs' diets may have affected the results, future studies should aim to control or record dietary intake.

Response: We thank the reviewer for the positive evaluation and for this constructive suggestion. We agree completely that the sample size is a key limitation and that this point should be stated more clearly and directly in the Discussion section.

In response to this suggestion, we have revised the opening of the limitations paragraph to more prominently feature the small sample size, dietary influence and to explicitly mention its effect on the generalizability of our findings.

Please see the revised text below:

The present study has several limitations. The primary limitation is the small sample size (n=17), which restricts the statistical power and the generalizability of our findings to a broader population of dogs with pulmonary hypertension. A larger study is necessary to confirm the encouraging clinical trends observed and to detect more subtle differences in other measurements. Secondly, this study relied on owner-reported scores to assess clinical efficacy. Although our scoring system was adapted from the QUEST study [39] for clinical relevance, these outcomes remain inherently subjective. Future research should therefore complement these scores with objective functional endpoints, such as the 6-minute walk test (6MWT) [40], to provide more robust evidence of therapeutic benefit. Thirdly, re-examination schedules for both groups were delayed in some cases, which could have influenced the consistency of our measurements. Finally, and perhaps most significantly, the lack of detailed dietary information for the enrolled dogs is a key limitation. The baseline intake of omega-3 fatty acids (EPA and DHA) from their regular diets was unknown and could not be accounted for. This is a potential confounding variable that may have influenced the results. Therefore, we suggest that future studies should aim to either control this variable by feeding all participants a standardized diet or, at minimum, record detailed dietary histories to allow for a more precise evaluation of the supplement's effects.

Despite these limitations, this study provides preliminary evidence that PCSO-524 is a safe add-on therapy that does not interfere with standard treatment for pulmonary hypertension. The encouraging improvements in specific clinical scores warrant further investigation in a larger, long-term trial to more definitively assess the impact of PCSO-524 on quality of life and survival in dogs with cardiovascular diseases.

3. Biomarker use: The lack of significant change in NT-proBNP concentrations deserves further exploration or explanation, especially considering its role in heart disease monitoring.

Response: We have expanded the discussion section to provide a more thorough exploration of the NT-proBNP findings. The revised text now contextualizes the observed concentrations by comparing them to established values in asymptomatic and symptomatic mitral valve disease [15, 16]. We also propose that the static nature of the biomarker reflects a stabilized hemodynamic burden from sildenafil therapy and contrast this with the different pharmacological effects of pimobendan. Finally, we clarify that the assays used in our study and the comparator study are considered comparable, strengthening our new interpretation. The revised section provides a more nuanced explanation for the biomarker's behavior in this specific clinical context.

Please see the revised text below:

Regarding biomarkers, NT-proBNP concentrations did not change significantly in either group during the study, with all median values remaining consistently elevated above the 800 pmol/L reference level for cardiac disease (Table 3). We interpret this lack of change not as a treatment failure, but as a reflection of the chronic, stable nature of the underlying cardiac disease in this medically managed population. We hypothesize that the primary therapy, sildenafil, was effective in stabilizing the dogs' condition, resulting in persistently high but static NT-proBNP levels that reflect an ongoing, but no longer worsening, hemodynamic load.

To support this interpretation, the observed concentrations were notably higher than those typically reported for dogs with asymptomatic myxomatous mitral valve disease, yet lower than for those in active congestive heart failure [17-19]. This finding contrasts with reports of NT-proBNP decreasing after pimobendan treatment [4], a discrepancy we attribute to differing pharmacological actions rather than assay performance, as the assays used are comparable [32]. Therefore, our results suggest that in stable PH patients, NT-proBNP is a valuable indicator of underlying disease presence but may have limited utility for monitoring short-term therapeutic response.

4. Clarity of figures and tables: Ensure that all supplemental tables and figures referenced (e.g., Supplemental Table 3, Figure 1) are available and labeled clearly for reviewers and readers.

Response: We thank the reviewer for this important reminder. We have performed a thorough check of the entire manuscript and its accompanying files to ensure all figures and tables are correctly referenced and presented.

We have confirmed that all supplemental tables and figures mentioned in the text have been uploaded correctly and are clearly labeled with descriptive titles and legends. Furthermore, we have double-checked that all in-text citations (e.g., "Figure 1," "Supplemental Table 3") correspond accurately to the correct items to ensure a clear and seamless experience for both reviewers and readers.

5. Minor language editing: Some grammatical and typographical errors should be addressed to improve the overall clarity and professionalism of the manuscript.

Response: We thank the reviewer for this reminder. We have performed a final, meticulous proofread of the entire manuscript to correct any remaining typographical and grammatical errors. We are confident the language is now clear, professional, and meets the high standards required for publication.

*Response for Reviewer #2*

1. The manuscript would benefit from a structured abstract.

Response: We thank the reviewer for this suggestion. Per the journal's author guidelines, which specify a single, non-structured paragraph for the abstract, we have maintained this format. However, based on feedback from Reviewer #3, we have completely rewritten the abstract to improve its clarity, structure, and flow, ensuring the objective, methods, results, and conclusions are presented more clearly within the required format.

2. It is recommended to crate a section for the Ethical considerations. Under Ethical considerations section, it is required to provide more information in the standard format.

Response: We thank the reviewer for highlighting the importance of clearly stating the ethical approval. In accordance with *PLOS ONE*'s specific formatting guidelines, we have now added our full ethics statement as the final subsection within the 'Materials and Methods' section of the manuscript, rather than creating a new standalone section. The added text reads as follows:

“All protocols in this study were approved by the Faculty of Veterinary Science, Mahidol University-Institute Animal Care and Use Committee, Thailand (COA No. MUVS-2018-06-32), in compliance with the Animals for Scientific Purposes Act, B.E. 2558 (A.D. 2015), Kingdom of Thailand. Written informed consent was obtained from the owners for the participation of their animals in this study.”

3. I recommend creating a new section for the study limitations.

Response: We appreciate the reviewer's suggestion to ensure the study limitations are clearly visible. According to standard manuscript structure and *PLOS ONE*'s guidelines, the limitations of a study are addressed within the 'Discussion' section. We have included a detailed paragraph dedicated to these limitations in our revised Discussion, ensuring it is clearly delineated and easy for the reader to identify. We believe this placement follows the journal's required format.

4. It is advised that a new section explaining any acronyms used in the article be added at the end, following the conclusion section.

Response: We thank the reviewer for this advice. In line with *PLOS ONE*'s style guide, we have opted not to include a separate list of abbreviations. Instead, we have carefully reviewed the entire manuscript to ensure that every acronym is defined in full upon its first use in both the abstract and the main text (e.g., 'Pulmonary Hypertension (PH)'). We believe this standard approach adheres to the journal's formatting requirements and ensures clarity for the reader throughout the text.

*Response for Reviewer #3*

Response overall: We are very grateful to Reviewer #3 for their exceptionally thorough and constructive review of our manuscript. We agree with the assessment that while the study's design and data are sound, the manuscript required significant revisions to improve its clarity, flow, and overall scholarly tone. We have undertaken a major revision of the entire manuscript based on these detailed recommendations, and we believe the paper is substantially stronger as a result. Below, we address each of the reviewer's points in detail.

1. Abstract: "While the abstract contains the necessary scientific content, it currently lacks the clarity and brevity expected by *PLOS ONE*. The sentences are overly long and dense, obscuring the main findings. I recommend reducing redundancy, improving flow, and focusing on clear communication of the objective, methods, and outcomes in under 300 words."

Response: We thank the reviewer for this important feedback. We agree that the original abstract was dense and did not communicate our findings with optimal clarity. Following the reviewer's suggestions, we have completely rewritten the abstract to improve its brevity, flow, and focus. The revised version clearly outlines the objective, methods, key results, and their significance in a coherent narrative under the 300-word limit.

2. Introduction : The Introduction provides a reasonable overview of pulmonary hypertension (PH) in dogs, including its causes, clinical manifestations, and current treatment options such as sildenafil. It also introduces PCSO-524 and its known anti-inflammatory properties. However, the section could be improved in several ways. The transitions between ideas are somewhat abrupt, and the research gap—why adjunctive therapy is needed despite standard treatment—is not clearly articulated. The mechanistic discussion of PCSO-524's effects is overly detailed and would benefit from being more concise and clinically focused. Additionally, there are several grammatical and formatting issues (e.g., “outcomesin” in line 75) that require correction. I recommend clarifying the rationale for the study and tightening the prose to improve readability and focus.

Response: We agree with the reviewer's assessment. We have revised the Introduction to better articulate the research gap and the clinical rationale for seeking an adjunctive therapy for PH. The transitions between ideas have been smoothed, and the mechanistic discussion of PCSO-524 has been made more concise and clinically focused, as suggested.

3. Materials and Methods:

The methodology is overall well-designed and clearly reported. The study is a prospective, randomized, placebo-controlled trial with appropriate ethical approvals and informed consent. Interventions and clinical assessments, including echocardiography, electrocardiography, and biomarker analysis, are detailed and replicable. The inclusion criteria and statistical analysis are also appropriate. Blinding is reported and appears to be well-executed. However, additional clarification on the randomization sequence generation and allocation concealment would improve transparency. Minor grammatical issues are present but do not detract from methodological rigor.

Response: We thank the reviewer for the positive assessment of our study's design and methodological rigor. We agree completely that adding specific details on the randomization sequence generation and allocation concealment process improves the manuscript's transparency and reproducibility. We have now added texts (see below) to clarify exactly how this was performed.

Dogs enrolled in the study were randomly divided into two groups. Group 1 was supplemented with PCSO-524 and group 2 was supplemented with a placebo, in addition to conventional treatments and sildenafil at a dose of 1 mg/kg orally every 12 hours. The severity of PH was used as a blocking factor in the randomization process to ensure an equal distribution of severe cases between the groups. The randomization process was stratified using PH severity as a blocking factor to ensure a balanced distribution of severe cases. For each severity block, a unique randomization sequence was generated using a computer-based random number generator. Allocation was concealed using sequentially numbered, opaque, sealed envelopes prepared according to this sequence. To ensure blinding, the envelopes were labeled neutrally as containing either ‘Medication A’ or ‘Medication B’, which corresponded to the PCSO-524 and placebo capsules. Placebos were manufactured to be identical in appearance to PCSO-524. Sildenafil was administered by the time of initiation PCSO-524 or placebo. PCSO-524 or placebo was added according to the recommendation, loading dose at 1 soft gel capsule per dog twice a day for 2 weeks, then 1 soft gel capsule once a day for the rest of the study. Investigators, owners, and sponsors were blinded to treatment allocation. Unblinding was performed following analysis.

4. Results:

The Results section presents the findings in a structured order and uses appropriate statistical methods. The within-group and between-group comparisons are clearly reported with valid p-values and consistent formatting (mean ± SD and median [range] where appropriate). Significant improvements in tricuspid regurgitation pressure gradient (TRPG) and exercise tolerance in the PCSO-524 group are appropriately highlighted. However, the section would benefit from improved language clarity and consistency in terminology (e.g., repeated use of “visit” vs. “day”). Grammatical errors and typographical mistakes (e.g., “rricuspid” instead of “tricuspid”) should be corrected. The use of vague language such as “numerically different improvement” should be revised to more standard phrasing like “a non-significant trend.” Overall, the statistical validity is strong, but presentation quality needs refinement.

Response: We thank the reviewer for positive assessment of our statistical methods and the overall structure of our results. We also agree entirely with the reviewer that the presentation quality required refinement to match the strength of the data.

To address the excellent points raised, we have performed a thorough revision of the entire Results section. Specifically:

- Clarity and Structure: The section has been rewritten with clear subheadings (Study population and baseline characteristics, Between-group comparisons at follow-up, and Within-group changes over time) to improve readability and narrative flow.
- Consistent Terminology: As suggested, all time-point references have been standardized to "Day 0," "Day 28," and "Day 56" throughout the text for improved consistency and precision.
- Correction of Errors: The section has been carefully proofread, and all typographical and grammatical errors, including the specific example of "rricuspid" being corrected to "tricuspid," have been fixed.
- Precise Scientific Language: Vague phrasing has been revised for accuracy. As specifically recommended, the phrase "numerically different improvement" has been replaced with the standard scientific term "a non-significant trend" to describe the finding for exercise tolerance (P=0.06).

We believe these changes have significantly improved the clarity and professionalism of the Results section, and we are grateful for the reviewer's detailed guidance in enhancing its presentation.

5. Discussion and Conclusion;

The Discussion section provides a broad and generally balanced interpretation of the study’s findings, particularly the observed improvements in exercise tolerance and coughing among dogs receiving PCSO-524. The authors appropriately contextualize their results within the existing literature and acknowledge key limitations, including small sample size, re-examination delays, and the absence of dietary control for omega-3 intake. These admissions strengthen the scientific integrity of the discussion.

However, the section is significantly weakened by numerous grammatical errors, awkward phrasing, and lack of narrative focus. Several paragraphs contain redundancies or imprecise statements (e.g., “may could have biased”), and the flow between ideas is inconsistent. Additionally, some claims are repeated unnecessarily or not fully supported by detailed reasoning (e.g., comparisons of TRPG reductions). References to human studies are overused in places without drawing direct relevance to the canine population. A major language edit is required to improve clarity, eliminate repetition, and present a more coherent, professional narrative.

The Conclusion, while consistent with the overall findings, is underdeveloped. It lacks clarity, contains grammatical issues, and does not adequately emphasize the statistically significant clinical improvements observed in the PCSO-524 group. Furthermore, the final sentence regarding the variability of TRPG is valid but ends the manuscript on a vague note. The conclusion should be rewritten to provide a concise, confident summary of findings and a clear recommendation for future research.

Overall, while the interpretation of results is scientifically reasonable, a thorough rewrite of the Discussion and Conclusion sections is necessary to ensure clarity, accuracy, and proper scholarly tone.

Response: We are very grateful to the reviewer for this detailed and highly constructive critique. We wholeheartedly agree that while the scientific interpretation was reasonable, the Discussion and Conclusion sections required a major revision to improve clarity, focus, and scholarly tone.

Following the reviewer's guidance, we have performed a thorough rewrite of both the Discussion and Conclusion.

- Narrative Focus and Clarity: We have restructured the Discussion to present a more logical and coherent narrative. The text now begins with the primary positive findings, followed by a focused interpretation of each key result (clinical scores, TRPG, biomarkers) with improved transitions between ideas.
- Elimination of Redundancy and Imprecision: We have performed a major language edit to eliminate redundancies, awkward phrasing, and imprecise statements. The reasoning for each claim has been clarified.
- Use of References: We have reviewed the use of references to human studies to ensure that their relevance to the canine context is more clearly and directly drawn.
- Rewritten Conclusion: The Conclusion has been completely rewritten to be more confident, concise, and impactful. It now starts by stating the primary finding, emphasizes the statistically significant clinical improvements, and ends with a clear and strong recommendation for future research, as suggested.

We believe these extensive revisions have addressed all the concerns raised and have significantly improved the quality, clarity, and impact of the manuscript's final sections. We thank the reviewer for helping us to so substantially strengthen our paper.

*Response for Reviewer #4*

This study is well-executed and provides valuable insights into adjunctive therapy for canine PH. Minor improvements in methodological transparency and discussion depth would enhance its impact.

Response: We sincerely thank the Reviewer for your positive and encouraging assessment of our work. We are grateful for the comments that our study is "well-executed" and provides "valuable insights."

We have carefully considered the suggestions for improvement and have performed a significant revision of the manuscript, which we believe addresses the points raised:

- Methodological Transparency: To enhance transparency, and in line with feedback from another reviewer, we have expanded the Materials and Methods section. We have now included precise details on the randomization sequence generation (specifying the use of a computer-based random number generator for each severity block) and the allocation concealment process (detailing the use of sequentially numbered, opaque, sealed envelopes). We believe this directly improves the transparency of our methodology as suggested.
- Discussion Depth: To enhance the depth of the Discussion, we have undertaken a major revision of this section based on the reviewers' collective feedback. This includes a more thorough exploration of our biomarker (NT-proBNP) findings and a complete rewrite of the Discussion and Conclusion to improve the narrative focus, clarify the interpretation of our results, and strengthen the final conclusions. We are confident that these revisions have added the depth and clarity the reviewer suggested would enhance the manuscript's impact.

We appreciate the reviewer's valuable guidance, which has helped us improve the overall quality of our paper.

*Response for Reviewer #5*

No additional comments.

Response: We thank the Reviewer for your time and for reviewing our manuscript.

*Response for Reviewer #6*

Dear Authors, This is a good manuscript and deserves to be published, however there are a few areas for improvement before the manuscript can be accepted.

Response: We sincerely thank the Reviewer for your positive evaluation and for providing clear, actionable feedback to improve our manuscript. We agree with the points raised and have performed a significant revision to address each one. We believe these changes have greatly enhanced the manuscript's impact and clarity.

1. Sample Size is too small. I suggest to label the title to reflect that aspect. You can call it a pilot study or something like that to justify the small sample.

Response: We thank the reviewer for this thoughtful suggestion. We agree completely that the small sample size is a primary limitation of our study and that this must be made clear to the reader. To ensure full transparency regarding this point, we have thoroughly expanded the limitations section within the Discussion to explicitly address the small sample size and its impact on statistical power and the generalizability of our findings.

Regarding the specific suggestion to label the study as a "pilot study" in the title, we have respectfully chosen to retain the original title. Our reasoning is that the term "pilot study" can sometimes imply a more preliminary investigation into feasibility or an open-label design. Given that our study employed a rigorous prospective, randomized, double-blind, placebo-controlled design with pre-specified endpoints, and successfully yielded statistically significant results for key clinical outcomes, we feel that a direct title is appropriate for the work as conducted.

We are confident that our detailed discussion of the limitations within the manuscript now effectively frames the study's scope for the reader, fulfilling the important intention behind the reviewer's valuable suggestion.

2. The diet is not controlled or recorded. This could significantly influence the study outcomes as it is particularly important when evaluating a nutraceutical intervention. I suggest you acknowledge this more explicitly and, if possible, include retrospective dietary assessments.

Response: We agree that the uncontrolled diet is a key limitation in a nutraceutical study. We have expanded our limitations section to more explicitly discuss this issue and its potential influence on the results. While a retrospective dietary assessment is an excellent suggestion, we determined it was not feasible to collect accurate and reliable dietary histories from the owners long after the study's completion. Instead, we have strongly recommended that future studies incorporate prospective dietary recording or use a standardized diet, as the reviewer suggests.

3. Study Group 1 (PCSO-524) had significantly worse baseline coughing scores. While this may partially explain the observed improvement, it could also bias the results. I suggest that you consider conducting an ANCOVA adjusting for baseline coughing scores.

Response: This is an excellent statistical point. While an ANCOVA is an ideal method, we have focused our interpretation on the analysis of the change in scores between follow-up visits, which helps account for individual starting points. We have also added a specific acknowledgment in the Discussion that the baseline imbalance is a limitation and that our findings, while supportive of a treatment effect, require confirmation in future studies with balanced groups.

4. There is a basic need for defining and expressing the lack of objective and functional endpoints. As the study relies heavily on owner-reported outcomes, it makes it even more important to use more objective data as that will for sure strengthen the findings and improve the quality of the study. You can also discuss this limitation and how further research studies can address it.

Response: We thank the reviewer for this important and constructive point. We agree completely that acknowledging the study's reliance on owner-reported outcomes and discussing the importance of objective endpoints is crucial for a balanced and transparent discussion.

To address this, we have added a dedicated paragraph to the "Limitations" section of our Discussion. This new text explicitly states that owner-reported scores are inherently subjective and can be influenced by a caregiver placebo effect. Furthermore, as suggested, we now discuss how future research would be significantly strengthened by complementing these scores with objective functional endpoints, specifically mentioning the 6-minute walk test (6MWT) as a key example.

5. Kindly elaborate on the implications of the lack of change in NT-proBNP concentrations.

Response: We thank the reviewer for this important suggestion. We agree that this finding required a more detailed discussion and interpretation. To address this, we have significantly expanded this section of the Discussion. Our revised text now provides a thorough exploration of the static NT-proBNP levels. Specifically, we propose a hypothesis that the stable, elevated levels reflect a stabilized hemodynamic burden due to the primary sildenafil therapy, rather than a treatment failure. We contextualize the observed values by comparing them to established levels in different stages of canine cardiac disease and contrast our findings with studies involving drugs with different pharmacological actions (e.g., pimobendan) to explain the discrepancy. Finally, we conclude with the clinical implication that for stable PH patients, NT-proBNP may be more valuable for confirming disease presence than for monitoring short-term therapeutic response.

6. Please elaborate the discussion on the composition and other synergistic actions of PCSO-524.

Response: We thank the reviewer for this excellent suggestion to add more depth to our discussion. We agree that elaborating on the unique nature of PCSO-524 is important for interpreting our findings. We have now added a paragraph to the Discussion that details the complex composition of PCSO-524 and discusses the potential for synergistic actions among its various lipid components, which may distinguish its effects from those of standard omega-3 supplements.

7. Ensure all abbreviations are defined at first mention in both the abstract and main text.

Response: We thank the reviewer for this essential reminder. We have performed a careful proofread of the entire manuscript and have ensured that all abbreviations (e.g., PH, MMVD, PCSO-524, TRPG) are fully defined in parentheses upon their first use. As requested, this check was performed independently for both the abstract and the main text to ensure clarity for the reader.

8. Ensure that grammatical errors are corrected before submission and that the English is up to the standards of the journal and general scientific literature.

Response: We thank the reviewer for this essential reminder. We have performed a comprehensive and meticulous review of the entire manuscript to address this point. The text has been thoroughly proofread by all co-authors to correct any grammatical errors, improve sentence structure, and ensure the language is clear, professional, and meets the high standards of scientific literature.

*Response for Reviewer #7*

This is a well written and interesting paper. It has great clinical application. There is so little available for this disease so it good to see some research on a natural product that will be helpful for most patients.

The statistical analysis appears to be correct and appropriate.

The data set presented is complete.

The English is appropriately written.

The references contain all the appropriate literature and does include both human and veterinary literature.

On line 273 results is spelled incorrectly.

Response: We are sincerely grateful to the Reviewer for your overwhelmingly positive and encouraging feedback on our manuscript. We are particularly pleased that the reviewer found our study to be well-written, interesting, and to have great clinical application. We also thank the reviewer for their careful reading and for catching the typographical error. The spelling of "results" has been corrected in the revised manuscript. We appreciate your supportive assessment of our work.

*Response for Reviewer #8*

The manuscript presents a prospective, randomized, double-blind, placebo-controlled study assessing the efficacy of *Perna canaliculus* oil (PCSO-524) as an add-on therapy to sildenafil in dogs with pulmonary hypertension (PH). The subject is novel and relevant, particularly as interest in nutraceuticals grows. The methodology is generally appropriate, and the manuscript is well structured. However, several issues—particularly related to small sample size, lack of dietary control, and interpretation of clinical significance—should be addressed before consideration for publication.

Response: We sincerely thank the Reviewer for your thorough and highly constructive review. We are encouraged by your assessment that the study is "novel and relevant" and "well structured." We agree with all the points raised regarding areas for improvement and, as recommended, have performed a major revision of the manuscript to address each concern. We agree completely that these are the key limitations of our study. To address them comprehensively, we have rewritten the Discussion and Conclusion to soften the tone, interpret all findings with appropriate caution, and explicitly recommend replication in a larger cohort.

Significantly expanded the Limitations section to provide a transparent and detailed discussion of these issues.

2. Methodology:

Design: Randomized, placebo-controlled design is appropriate.

Response: Thank you very much.

Blinding: Adequately blinded (owners, investigators, and sponsors), which strengthens the credibility of the findings.

Response: Thank you very much.

Sample Size: Only 17 dogs completed the study, which severely limits statistical power.

Response: We agree completely that the small sample size is a primary limitation of our study and that this must be made clear to the reader. To ensure full transparency regarding this point, we have thoroughly expanded the limitations section within the Discussion to explicitly address the small sample size and its impact on statistical power and the generalizability of our findings.

Inclusion Criteria: Well-defined, but would benefit from clearer exclusion criteria (e.g., how arthritis was ruled out).

Response: We have now added a sentence to the Materials and Methods to clarify this. While a full orthopedic exam was not part of the screening, we have clarified that dogs receiving specific ongoing medication for orthopedic disease (e.g., NSAIDs) were excluded to avoid confounding analgesic effects. We have also expanded on this in the Discussion, acknowledging that subclinical osteoarthritis could be a confounding factor for the exercise tolerance outcome.

Nutritional Confounding: No documentation of dietary intake or background EPA/DHA content, which critically affects interpretation of omega-3 related outcomes.

Response: We agree that the uncontrolled diet is a critical limitation. As suggested, we have clearly and explicitly stated this limitation in the Discussion. While creating a table of baseline diets is a valuable suggestion, it was not feasible to collect this information accurately and retrospectively. We have instead highlighted this as a critical area for improvement in future research, recommending either the use of a standardized diet or prospective dietary recording.

Suggestions for Revision:

Include a table summarizing baseline diets or clearly state the limitation due to dietary variability.

Response: It was not feasible to collect this information accurately and retrospectively. We have instead highlighted this as a critical area for improvement in future research, recommending either the use of a standardized diet or prospective dietary recording. We added in limitation of the study as the text below:

“Finally, and perhaps most significantly, the lack of detailed dietary information for the enrolled dogs is a key limitation. The baseline intake of omega-3 fatty acids (EPA and DHA) from their regular diets was unknown and could not be accounted for. This is a potential confounding variable that may have influenced the results. Therefore, we suggest that future studies should aim to either control this variable by feeding all participants a standardized diet or, at minimum, record detailed dietary histories to allow for a more precise evaluation of the supplement's effects.”

Clarify how orthopedic comorbidities were ruled out (as they may influence exercise tolerance).

Include a table summarizing baseline diets or clearly state the limitation due to dietary variability.

Response: We have now added a sentence to the Materials and Methods to clarify this. “While a full orthopedic exam was not part of the screening, we have clarified that dogs receiving specific ongoing medication for orthopedic disease (e.g., NSAIDs) were excluded to avoid confounding analgesic effects.” We have also expanded on this in the Discussion, acknowledging that subclinical osteoarthritis could be a confounding factor for the exercise tolerance outcome.

3. Statistical Analysis:

Suggestion: Provide confidence intervals alongside p-values to support effect estimates.

Response: We sincerely thank the reviewer for this excellent and important statistical suggestion. We wholeheartedly agree that reporting confidence intervals alongside effect estimates represents the gold standard for robust statistical analysis, as they provide invaluable insight into the precision of the findings.

In considering how to best implement this for our manuscript, we revisited our statistical approach. As detailed in our methods, the nature of our data required us to use a combination of parametric and non-parametric tests, based on the results of our normality testing (Shapiro-Wilk test). Consequently, our analyses include the Independent T-test and Repeated Measures ANOVA, but also non-parametric tests such as the Mann-Whitney U, Friedman, and Chi-square tests.

While confidence intervals for mean differences are readily available for t-tests, they are not a standard output for the non-parametric tests used in our statistical software. We felt that providing CIs for only a subset of our results would create an inconsistent and potentially confusing reporting style for the reader.

Therefore, to ensure our reporting remains as clear, consistent, and transparent as possible across all analyses, we have focused on providing comprehensive descriptive statistics (means ± SD for normally distributed data; medians and interquartile ranges for non-normal data) along with the exact p-values for all our primary outcomes.

We believe this detailed presentation allows the reader to fully assess the data distribution, the magnitude of the effects, and their statistical significance. We hope this approach is satisfactory, and we are very grateful for the reviewer's guidance in helping us to present our statistical findings as transparently as possible.

5. Interpretation and Discussion:

The discussion is thorough and references relevant literature.

However, the role of confounding variables (e.g., underlying osteoarthritis, nutrition) is under-emphasized.

The statement that PCSO-524 improved coughing and exercise tolerance should be softened to reflect the exploratory nature of this pilot study.

Suggestion:

Revise tone to reflect preliminary nature and acknowledge limitations more explicitly.

Consider recommending replication in a larger, multicenter cohort.

Response: We thank the reviewer for your positive feedback on our discussion and for your insightful comment regarding the emphasis on confounding variables. We agree that a more thorough discussion of the potential impact of these factors is essential for a balanced interpretation of our findings. To address this, we have performed a major revision on

- Role of Osteoarthritis: We have performed revisions in both the Materials and Methods and the Discussion to more strongly emphasize and clarify the potential confounding role of underlying orthopedic disease. In the Materials and Methods, we have now specified that while a full orthopedic exam was not part of the screening protocol, dogs receiving specific ongoing medication for orthopedic disease (e.g., NSAIDs) were excluded to avoid confounding analgesic effects. Building on this, the Discussion now gives greater emphasis to the possibility that the observed improvement in exercise tolerance may be partially attributable to the known analgesic effects of PCSO-524 on subclinical osteoarthritis, which is a common comorbidity in this elderly patient population.
- Role of Nutrition: We have significantly expanded the Limitations section to explicitly identify the lack of dietary control as a key confounding variable and have discussed its potential influence on the study's outcomes. We also suggest that future studies should aim to either control this variable by feeding all participants a standardized diet or, at minimum, record detailed dietary histories to allow for a more precise evaluation of the supplement's effects.”

Final Recommendation:

Major Revision

Response: We are very grateful to the reviewer for their insightful comments and their recommendation for a major revision. We found the feedback extremely helpful and have undertaken a complete revision of the manuscript to address all concerns. We believe the paper's clarity and scientific integrity have been significantly enhanced as a result of this guidance.

Summary of Required Revisions:

Address Small Sample Size Limitations in the Discussion and interpret findings with appropriate caution.

Response: We thank the reviewer for highlighting this crucial point. We agree completely that the small sample size is a primary limitation that necessitates a cautious interpretation of our findings. To address this thoroughly throughout the manuscript, we have undertaken several key revisions:

- Expanded Limitations: We have significantly expanded the limitations section within the Discussion. It now explicitly details how the small sample size (n=17) restricts the study's statistical power and the generalizability of the results to a broader clinical population.
- Cautious Interpretation and Tone: The tone throughout the entire Discussion and Conclusion has been carefully moderated. We have softened our claims, framing the positive results as "encouraging trends" that "warrant further investigation" rather than as definitive proof of efficacy.

We are confident that this multi-faceted approach ensures that our findings are presented and interpreted with the appropriate caution that the reviewer rightly recommended. We are grateful for the guidance.

Include or Discuss Diet Control and how this may have influenced omega-3 intake.

Response: We agree that the uncontrolled diet is a critical limitation. As suggested, we have clearly and explicitly stated this limitation in the Discussion. While creating a table of baseline diets is a valuable suggestion, it was not feasible to collect this information accurately and retrospectively. We have instead highlighted this as a critical area for improvement in future research, recommending either the use of a standardized diet or prospective dietary recording.

Clarify and Justify Exclusion of Orthopedic Comorbidities or acknowledge their potential confounding effects.

Response: We have now added a sentence to the Materials and Methods to clarify this. “While a full orthopedic exam was not part of the screening, we have clarified that dogs receiving specific ongoing medication for orthopedic disease (e.g., NSAIDs) were excluded to avoid confounding analgesic effects.” We have also expanded on this in the Discussion, acknowledging that subclinical osteoarthritis could be a confounding factor for the exercise tolerance outcome.

Correct Minor Language Errors and Typos.

Response: We thank the reviewer for this reminder. We have performed a final, meticulous proofread of the entire manuscript to correct any remaining typographical and grammatical errors. We are confident the language is now clear, professional, and meets the high standards required for publication.

Soften Conclusions to reflect the exploratory nature of the findings.

Response: We agree completely with this crucial point. To ensure our interpretation accurately reflects the nature of our study, we have undertaken several key revisions:

1) The tone throughout the Discussion and Conclusion has been carefully moderated. We now frame the results as "encouraging trends" that "warrant further investigation," rather than as definitive proof of efficacy.

2) The Conclusion itself has been completely rewritten to provide a concise summary of the statistically significant findings while emphasizing that they are preliminary and require confirmation in larger, more definitive trials.

We believe these comprehensive changes ensure that our findings are interpreted with the appropriate caution suggested by the reviewer. We appreciate the opportunity to improve our manuscript with this valuable feedback.

*Response for “MS word file name review report”*

Intro

Line 54: PH specific ( spelling mistake)

Line 290: the pancutation is not correct

Line 162: use the both groups instead of 2 groups.

Response: We thank the reviewer for their careful and detailed proofreading of our manuscript. We appreciate them for pointing out these specific errors, which help improve the overall quality of the paper.

We have corrected all the noted items in the revised manuscript as follows:

- Line 54: The spelling error for "PH-specific" has been corrected.
- Line 290: The punctuation has been corrected for clarity.
- Line 162: As suggested, the phrasing has been changed from "2 groups" to "both groups."

We are grateful for the reviewer's help in polishing our manuscript.

Methods:

-The randomization process could be described more precisely, including how subjects were allocated.

Response: We thank the reviewer for the positive assessment of our study's design and methodological rigor. We agree completely that adding specific details on the randomization sequence generation and allocation concealment process improves the manuscript's transparency and reproducibility. We have now added texts (see below) to clarify exactly how this was performed.

Dogs enrolled in the study were randomly divided into two groups. Group 1 was supplemented with PCSO-524 and group 2 was supplemented with a placebo, in addition to conventional treatments and sildenafil at a dose of 1 mg/kg orally every 12 hours. The severity of PH was used as a blocking factor in the randomization process to ensure an equal distribution of severe cases between the groups. The randomization process was stratified using PH severity as a blocking factor to ensure a balanced distribution of severe cases. For each severity block, a unique randomization sequence was generated using a computer-based random number generator. Allocation was concealed using sequentially numbered, opaque, sealed envelopes prepared according to this sequence. To ensure blinding, the envelopes were labeled neutrally as containing either ‘Medication A’ or ‘Medication B’, which corresponded to the PCSO-524 and placebo capsules. Placebos were manufactured to be identical in appearance to PCSO-524. Sildenafil was administered by the time of initiation PCSO-524 or placebo. PCSO-524 or placebo was added according to the recommendation, loading dose at 1 soft gel capsule per dog twice a day for 2 weeks, then 1 soft gel capsule once a day for the rest of the study. Investigators, owners, and sponsors were blinded to treatment allocation. Unblinding was performed following analysis.

-The data collection points (days 0, 28, and 56) are clearly stated, but justification for the choice of time intervals could be added.

Response: We thank the reviewer for this excellent suggestion to improve the clarity of our study design. We have now added the justification for these time intervals to the Materials and Methods section.

Our rationale is as follows: The follow-up intervals of day 28 and day 56 were selected based on a combination of clinical and practical considerations. Please see the texts added: “The follow-up intervals of day 28 and day 56 were selected based on a combination of clinical and practical considerations. A one-month follow-up (day 28) represents a standard re-examination point for chronic conditions and was considered a sufficient period to observe potential initial effects from the nutraceutical supplement. The second follow-up at day 56 was planned to assess the sustained effects of the intervention over a more prolonged period.”

We also acknowledge in the manuscript that minor logistical delays in these re-examination timings occurred. The actual timings are reported transparently in the Results, and the potential impact of these delays is discussed in the Limitations section. We believe this clarification of our intended schedule strengthens the methodological description.

-It would be beneficial to provide more details on how questionnaire reliability was ensured, particularly in assessing clinical signs reported by owners.

Response: We thank the reviewer for this important question regarding the reliability of the owner-reported outcomes. To address this directly within the context of our findings, we have added a dedicated paragraph to the main body of the limitation of the study in the Discussion.

This clarifies that our scoring system was adapted from the questionnaire used in the QUEST study to provide a structured and clinically relevant foundation. It also transparently acknowledges that these owner-reported outcomes are inherently subjective. Finally, as suggested, it discusses how future studies can be strengthened by complementing these scores with objective functional endpoints, such as the 6-minute walk test (6MWT).

We believe this integrated approach provides a more thorough and critical interpretation of our results.

Discussion:

-The lack of dietary reporting is appropriately noted but could be expanded with a recommendation for future studies to standardize omega-3 intake.

Response: We agree that the uncontrolled diet is a critical limitation. As suggested, we have clearly and explicitly stated this limitation in the Discussion. While creating a table of baseline diets is a valuable suggestion, it was not feasible to collect this information accurately and retrospectively. We have instead highlighted this as a critical area for improvement in future research, recommending either the use of a standardized diet or prospective dietary recording.

-The neutrophil-to-lymphocyte ratio and RDW findings are interesting but require a stronger clinical interpretation—whether these changes might have unintended consequences long-term

Response: We thank the reviewer for this insightful comment, and we agree that these unexpected findings require a stronger clinical interpretation and a discussion of potential long-term implications. We have revised this section of the Discussion to address this directly. The revised text now:

- Frames the finding as a "clinical paradox" (i.e., clinical improvement despite changes in typically negative prognostic markers).
- Reinforces that all values remained within the normal reference range, which mitigates immediate clinical concern.
- Explicitly acknowledges that given the small sample size, these isolated statistical findings could be incidental.
- Directly addresses the reviewer's point by stating that unintended long-term consequences cannot be dismissed and that this is a critical area for future monitoring.
- Concludes with a strong recommendation that future, larger studies must specifically track these hematological markers to determine their long-term significance and confirm the supplement's safety profile.

We believe this expanded discussion now provides the stronger, more critical interpretation that the reviewer rightly suggested.
