# Supplementary material for: Clinical improvement in canine pulmonary hypertension with Perna canaliculus oil (PCSO-524) add-on therapy: Effects on exercise tolerance and cough
Source: PLoS One. 2025 Sep 29;20(9):e0333526. doi: 10.1371/journal.pone.0333526 (PMC12478914; doi:10.1371/journal.pone.0333526)
Supplement: Attachment — Submitted filename: Response to editor and reviewer.docx [file pone.0333526.s013.docx]

**Journal Requirements:**

If the reviewer comments include a recommendation to cite specific previously published works, please review and evaluate these publications to determine whether they are relevant and should be cited. There is no requirement to cite these works unless the editor has indicated otherwise. 

Please review your reference list to ensure that it is complete and correct. If you have cited papers that have been retracted, please include the rationale for doing so in the manuscript text, or remove these references and replace them with relevant current references. Any changes to the reference list should be mentioned in the rebuttal letter that accompanies your revised manuscript. If you need to cite a retracted article, indicate the article’s retracted status in the References list and also include a citation and full reference for the retraction notice.

Response: We confirm that we have adhered to all journal requirements for the reference list:

- We have reviewed our reference list to ensure it is complete and correct.
- We have verified that no cited articles have been retracted.
- All changes to the reference list made during this revision are noted in this rebuttal letter."

We have modified author contribution (add Nattapon Riengvirodkij to formal analysis, and Walasinee Sakcamduang to writing original draft).

We have also undertaken further revisions to enhance the manuscript. The tables within the main document (Tables 1-4) have been modified for improved clarity and accuracy. Furthermore, the supplementary tables have been revised and are submitted as a separate file in accordance with journal guidelines.

**Additional Editor Comments:**

5. Is the manuscript presented in an intelligible fashion and written in standard English?

PLOS ONE does not copyedit accepted manuscripts, so the language in submitted articles must be clear, correct, and unambiguous. Any typographical or grammatical errors should be corrected at revision, so please note any specific errors here.

Reviewer #2: Yes

Reviewer #3: No

Reviewer #6: Yes

Response: We are grateful to the reviewers for their comments. In light of the feedback from Reviewer #3, we have performed a careful and thorough proofread of the entire manuscript. We have meticulously corrected all typographical and grammatical errors and revised sentences for clarity and precision. We believe the revised version now fully meets the journal's language requirements.

**Point-by-point response to each reviewer comment**

*Response for Reviewer #2 (file attached ‘Review 55a.pdf’)*

1. The manuscript would benefit from a structured abstract. The revised version has the same unstructured abstract as the primary submission.

Response: We thank the reviewer for this suggestion. Per the journal's author guidelines, which specify a single, non-structured paragraph for the abstract, we have maintained this format. We have completely written the abstract to improve its clarity, structure, and flow, ensuring the objective, methods, results, and conclusions are presented more clearly within the required format.

2. It is recommended to crate a section for the Ethical considerations. Under Ethical considerations section, it is required to provide more information in the standard format.
It is better presented in the revised version.

Response: Thank you very much.
3. I recommend creating a new section for the study limitations. Has not been addressed by authors.
Response: We appreciate the reviewer's suggestion to ensure the study limitations are clearly visible. According to standard manuscript structure and *PLOS ONE*'s guidelines, the limitations of a study are addressed within the 'Discussion' section. We have included a detailed paragraph dedicated to these limitations in our revised Discussion, ensuring it is clearly delineated and easy for the reader to identify. We believe this placement follows the journal's required format.

4. It is advised that a new section explaining any acronyms used in the article be added at the end, following the conclusion section. No action by authors.
Response: We thank the reviewer for this advice. In line with *PLOS ONE*'s style guide, we have opted not to include a separate list of abbreviations. Instead, we have carefully reviewed the entire manuscript to ensure that every acronym is defined in full upon its first use in both the abstract and the main text (e.g., 'Pulmonary Hypertension (PH)'). We believe this standard approach adheres to the journal's formatting requirements and ensures clarity for the reader throughout the text.

Thanks to the authors for their hard work on this Revision. It’s a pleasure to review such a
contribution to the field.

Response: Thank you very much for your positive response.
